# Linking demographic and food‐web models to understand management trade‐offs

**DOI:** 10.1002/ece3.5385

**Published:** 2019-07-17

**Authors:** Martina Kadin, Morten Frederiksen, Susa Niiranen, Sarah J. Converse

**Affiliations:** ^1^ School of Aquatic and Fishery Sciences University of Washington Seattle Washington USA; ^2^ Swedish Museum of Natural History Stockholm Sweden; ^3^ Department of Bioscience Aarhus University Roskilde Denmark; ^4^ Stockholm Resilience Centre Stockholm University Stockholm Sweden; ^5^ U.S. Geological Survey Washington Cooperative Fish and Wildlife Research Unit, School of Environmental and Forest Sciences (SEFS) and School of Aquatic and Fishery Sciences (SAFS) University of Washington Seattle Washington USA

**Keywords:** alcids, ecosystem‐based management, food‐web models, population models, seabirds, trophic cascades

## Abstract

Alternatives in ecosystem‐based management often differ with respect to trade‐offs between ecosystem values. Ecosystem or food‐web models and demographic models are typically employed to evaluate alternatives, but the approaches are rarely integrated to uncover conflicts between values. We applied multistate models to a capture–recapture dataset on common guillemots *Uria aalge* breeding in the Baltic Sea to identify factors influencing survival. The estimated relationships were employed together with Ecopath‐with‐Ecosim food‐web model simulations to project guillemot survival under six future scenarios incorporating climate change. The scenarios were based on management alternatives for eutrophication and cod fisheries, issues considered top priority for regional management, but without known direct effects on the guillemot population. Our demographic models identified prey quantity (abundance and biomass of sprat *Sprattus sprattus*) as the main factor influencing guillemot survival. Most scenarios resulted in projections of increased survival, in the near (2016–2040) and distant (2060–2085) future. However, in the scenario of reduced nutrient input and precautionary cod fishing, guillemot survival was projected to be lower in both future periods due to lower sprat stocks. Matrix population models suggested a substantial decline of the guillemot population in the near future, 24% per 10 years, and a smaller reduction, 1.1% per 10 years, in the distant future. To date, many stakeholders and Baltic Sea governments have supported reduced nutrient input and precautionary cod fishing and implementation is underway. Negative effects on nonfocal species have previously not been uncovered, but our results show that the scenario is likely to negatively impact the guillemot population. Linking model results allowed identifying trade‐offs associated with management alternatives. This information is critical to thorough evaluation by decision‐makers, but not easily obtained by food‐web models or demographic models in isolation. Appropriate datasets are often available, making it feasible to apply a linked approach for better‐informed decisions in ecosystem‐based management.

## 1 INTRODUCTION

Ecosystem‐based management has emerged as a promising approach to balance the diverse ways people use and modify marine systems (Curtin & Prellezo, 2010). Quantitative approaches are needed to assess ecosystem effects of management alternatives (Levin, Fogarty, Murawski, & Fluharty, 2009). Management of key ecosystem drivers, such as fisheries or eutrophication, is commonly evaluated with food‐web or ecosystem models. These models focus on groups or species of high ecological importance, while species occurring in lower numbers or with limited ecological function are rarely assessed. For iconic or controversial species or for populations of conservation concern, demographic models may be developed to assess management alternatives (Frederiksen, Lebreton, Pradel, Choquet, & Gimenez, 2014). It is rare that assessments merge insights from the two modeling approaches, despite the opportunity to uncover important trade‐offs associated with management alternatives and support conservation of less common species.

Including less common species in a food‐web or ecosystem model can be cumbersome. The increased complexity of interactions is a practical challenge while the limited data often translate to substantial uncertainty concerning relationships. End‐to‐end ecosystem models such as Atlantis are well suited to guide strategic direction setting, but evaluation of specific management decisions is hindered by inadequate precision (Fulton et al., 2011). Food‐web models that require a certain type of data, such as mass‐balance models like Ecopath‐with‐Ecosim (Christensen & Walters, 2004), may prohibit inclusion of specific species when relevant input data are lacking (but see, Lynam et al. (2017) for a food‐web model built on several types of time series) or provide results of limited relevance for migratory or long‐lived species about which information on demographic change, rather than biomass change, is needed to guide management.

Demographic models can provide detailed insights about population parameters and environmental variables affecting them, supporting decision‐making when management actions influence those variables directly (Frederiksen et al., 2014). Most management resources and efforts, however, are targeted toward broad‐scale drivers, such as harvest of commercially important species or nutrient input. Effects of management interventions may cascade through the food web and be amplified or counteracted by species interactions (Estes et al., 2011). Abiotic factors may further modify the influence management actions have on the ecological variables, for example, prey stock size, directly related to population parameters. To capture such effects, demographic models can usefully be linked to food‐web models.

**Figure 1:**
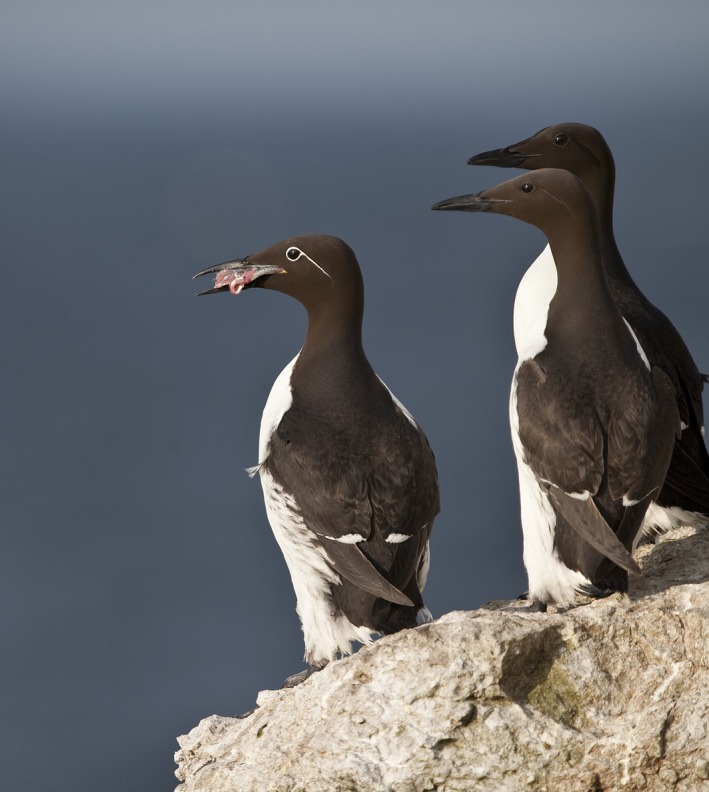
Common guillemot *Uria aalge*. Photograph: Aron Hejdström

Here, we demonstrate linking adult survival probability in common guillemots *Uria aalge* (hereafter guillemot, Figure 1), breeding in the Baltic Sea, with future scenarios for management of the main environmental drivers in the region, including Atlantic cod *Gadus morhua* fisheries and eutrophication. The guillemot has few alternative prey sources in the Baltic Sea, and studies suggest that sprat *Sprattus sprattus* is their main prey year‐round (Kadin, Österblom, Hentati‐Sundberg, & Olsson, 2012 and references therein). Abundance of sprat increased dramatically during the 1990s following the collapse of its main predator, cod of the eastern Baltic stock. Declines in cod and subsequent increases in sprat were part of an ecosystem regime shift caused by high cod fishing pressure in combination with eutrophication effects and changes in climate (Möllmann et al., 2009). Effects cascading through the food web included lower condition and weight‐at‐age of sprat, due to high intraspecific food competition (Casini et al., 2011), which reduced the energy content, and thereby quality, of sprat as prey for chick‐rearing guillemots (Kadin et al., 2012; Rojbek, Tomkiewicz, Jacobsen, & Stottrup, 2014). Sprat quality as well as quantity could potentially impact guillemot adult survival, along with direct and indirect effects of climate. Further, the historical pattern suggests that alternatives for managing regional drivers, mainly cod fishing and eutrophication, can result in indirect effects on guillemots mediated through the food web. Understanding these effects is relevant, not least for evaluating ongoing efforts to reduce nutrient input to lower levels under the Baltic Sea Action Plan and restore the eastern Baltic cod stock, concurrent with biodiversity conservation commitments (HELCOM, 2007, 2018; ICES, 2013).

To understand potential impacts of efforts currently under implementation and alternative scenarios, we analyze variables influencing guillemot survival and project the near (2016–2040) and distant (2060–2085) future impacts on survival under six scenarios. We predict sprat quantity to be the variable with strongest influence on guillemot survival, resulting in lower survival in future scenarios with a reduced sprat stock. Including two levels of cod fishing pressure and three levels of nutrient input, our scenarios account for the key anthropogenic drivers of ecological change in the Baltic Sea. The drivers do not have known direct effects on guillemots, but are the foci of societal discussions and decision‐making bodies (BalticSTERN, 2013; BirdLife Europe et al., 2015; Coalition Clean Baltic, Oceana, & The Fisheries Secretariat, 2013; HELCOM, 2007, 2013). The discussions rarely include the consideration of indirect effects that may result in conflicts with other management objectives, such as conservation. We specifically aim to explore the potential for management trade‐offs, manifested as likely negative impacts on the guillemot population, from management alternatives that are otherwise favored by decision‐makers.

Our work illustrates how demographic models can be linked to food‐web models to describe likely population trends under different management scenarios and climate change. Particularly, it showcases a way to detect impacts on less abundant species when management targets broad‐scale drivers. We discuss applications and potential extensions of this approach as a tool in ecosystem‐based management and conservation.

## 2 MATERIALS AND METHODS

### 2.1 Case study

Guillemots are long‐lived seabirds with a circumpolar distribution. Onset of reproduction is delayed, and birds typically start breeding when 4 or 5 years old, raising a maximum of one chick per year. The Baltic Sea population has increased in abundance through most of the 20th century (Olsson & Hentati‐Sundberg, 2017; Ottvall et al., 2009), and colonies were established in the Stockholm archipelago in the 1970s. The study colony is located on Kalken (19°30’ E, 59°26’N), an islet in the Svenska Högarna group, in the outermost part of the Stockholm archipelago. Ringing and recapture of guillemots has taken place once a year, with visits aimed to match the peak of the breeding season. This study made use of data from the 1,057 full‐grown birds ringed from 1995 to 2014. The majority of birds captured were likely to be breeding adults, but immature birds may be captured as well.

### 2.2 Survival models and covariates

We estimated annual survival probability of guillemots using a multistate model framework in E‐Surge 1.8.5 (Choquet, Rouan, & Pradel, 2009, see details in Appendix A). The multistate model allowed us to account for transitions of guillemots between breeding sites, specifically emigration from Kalken (state *Kalken*) to other breeding colonies (designated as state *Other*), which can otherwise bias survival estimates.

Model structure was informed by goodness‐of‐fit tests carried out in U‐Care 2.3.2 (Choquet, Lebreton, Gimenez, Reboulet, & Pradel, 2009). First, we checked recaptures at Kalken and *Other* with the multistate option. Test 3G.SR suggested a transience effect (χ^2^ = 58.9, *df* = 19, *p* ≪ 0.001). A p‐value was not available from the WBWA test, which we attributed to birds moving only from Kalken to *Other* in our model (see Appendix A). Remaining test components resulted in an overdispersion coefficient, $\hat{c}$ = 1.65. To examine model fit for observations at Kalken, hence ignoring emigration, we checked a subset of data, including only the (re‐)captures at Kalken, using the single‐state option. This also indicated the presence of transient individuals (Test 3.SR: χ^2^ = 74.9, *df* = 19, *p* ≪ 0.001). With a model including two “ringing age”‐classes to model transience, remaining overdispersion could be accounted for using $\hat{c}$ = 1.31 in a single‐state analysis. Based on these tests, we analyzed the data using two “ringing age”‐classes at Kalken and $\hat{c}$ = 1.5 to adjust model selection and estimates of precision. We also examined effects of higher $\hat{c}$ on model ranking (minor changes only, see Appendix A).

**Figure 2:**
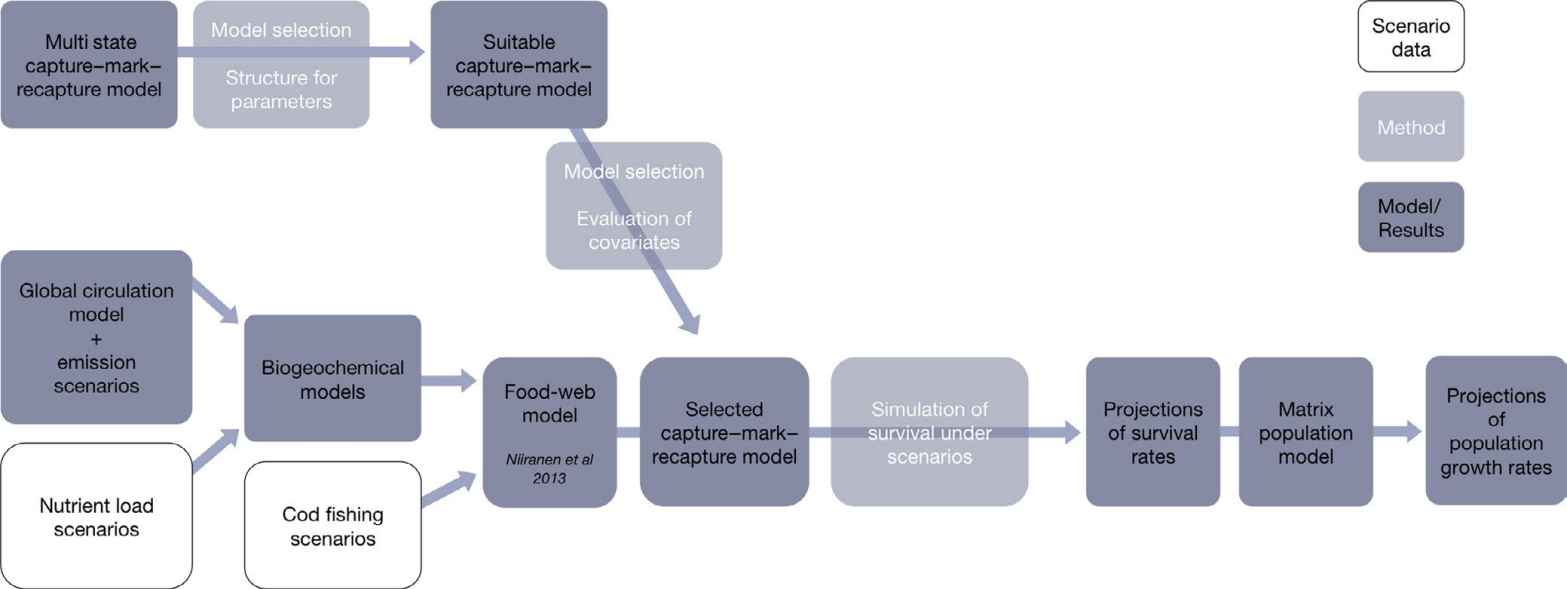
Conceptual overview of the modeling approach. The upper path illustrates the demographic model (for details see Section 2.2), and the lower path starts with the input to the food‐web model to show the construction and analysis of scenarios, specifically projections of survival and population growth rates following the merge of the path (see Section 2.3)

Model selection was based on QAICc (Akaike's information criterion corrected for lack of fit and sample size). We modeled parameters in stages, because of the large numbers of parameters considered, and therefore the large number of models we would have to implement if we were to evaluate all possible combinations. Model selection began with modeling survival probabilities, starting with structures of intermediate complexity for transition and detection probabilities. After having identified the most parsimonious structure for survival probabilities, we continued with transition probabilities. Last, we modeled detection probabilities, first exploring structures of time‐dependence for recapture probabilities, second time‐dependence for recovery probability, and third “age‐since‐ringing”‐dependence in recaptures at Kalken. At each stage, we cross‐checked the best model against competing models from the previous modeling stages to ensure that variation was appropriately apportioned among parameters. Having identified a suitable model structure, we evaluated relationships between survival and environmental covariates (Figure 2).

Prey covariates included Baltic Sea sprat abundance and spawning stock biomass (SSB), estimated at the beginning of each year, as well as a proxy for prey quality, the annual mean weight of four‐year‐old sprat based on samples from the commercial catch in the Baltic Sea (ICES, 2016). We used data for the entire Baltic Sea sprat population (ICES subdivisions 22–32), as ring recovery analyses indicate that the guillemots use a large part of the central Baltic Sea during winter (Fransson, Österblom, & Hall‐Karlsson, 2008; Österblom, Fransson, & Olsson, 2002), and preliminary results of geolocator (light‐logging devices) studies of the birds breeding at Kalken in particular (M. Kadin, unpubl data) also suggest that they are using the majority of the central Baltic Sea over the course of a year. Prey variables sampled at the same scale would thus provide the best match with overwinter survival.

Environmental factors at small and large scales may impact seabird survival. Regional climate is often represented by the North Atlantic Oscillation index during winter (December–March, wNAO; Omstedt et al., 2014), which may have a direct relationship with survival, thus with no time‐lags. The relationship can also be indirect when effects are mediated through the food web, often with a time‐lag of 1 year (Sandvik, Erikstad, Barrett, & Yoccoz, 2005). We used the Hurrell station‐based wNAO (Hurrell & National Center for Atmospheric Research Staff, 2017; Hurrell & Deser, 2010). Local conditions, such as sea surface temperature (SST) or ice cover, may have a stronger causal link to survival than regional climate, so we included central Baltic Sea SST and maximum sea ice extent, in addition to wNAO. The SST covariates were annual averages based on temperature measurements at depth <10 m from January–March in the area 54–60°E, 14–22°N, obtained from the SHARK database at the Swedish Meteorological and Hydrological Institute (SMHI). Annual maximum extent of sea ice in the Baltic Sea was based on the public climate indicator time series (SMHI, 2016). All three variables were modeled with no lags and 1‐year time‐lags.

We assessed the relationships between the survival of previously ringed birds at Kalken and each environmental covariate, using the highest ranked general model. The importance of covariates was determined using analysis of deviance (ANODEV; Skalski, Hoffman, & Smith, 1993). We confirmed that the results were robust to model selection uncertainty (see Table 4) by also fitting the identified effects to several other high‐ranking models (i.e., three models within 2 QAICc units, results not shown).

### 2.3 Scenario analysis

We used simulations of future scenarios from a central Baltic Sea food‐web model (Niiranen et al., 2013) to understand guillemot survival under different ecosystem management alternatives, as mediated through the sprat population. The time‐dynamic Ecosim model (Christensen & Walters, 2004) was developed to simulate the combined effects of climate, cod fishing pressure, and eutrophication on key components of the central Baltic Sea (Niiranen et al., 2013; Tomczak, Niiranen, Hjerne, & Blenckner, 2012). Climate change was incorporated by using three emission scenarios (A2, A1B, and A1B1) driving a global circulation model from which the results were dynamically downscaled by regional climate models (Meier et al., 2012). An ensemble of three Baltic Sea biogeochemical models was then driven by the resulting regional climate scenarios in combination with three regional nutrient input scenarios to produce time series of environmental drivers. The relevant environmental drivers were used to force the food‐web model in combination with two cod fishing scenarios (Niiranen et al., 2013, Figure 2).

This resulted in biomass projections of key Baltic Sea fish stocks under six scenarios: three levels of nutrient input (*Decrease*, which corresponds to adhering to the Baltic Sea Action Plan (HELCOM, 2007), *Reference*, and *Increase*) crossed with two fishing mortalities of cod (*Precautionary*, fishing mortality (*F*) = 0.3 following the last management plan (ICES, 2013), and *Intensive*, *F* = 1.1 corresponding to high exploitation). The resulting projections for sprat SSB averaged over climate scenarios and biogeochemical models are presented in Niiranen et al. (2013).

Scenario analyses were conducted in R 3.3.2 (R Core Team, 2016). The relationship between survival and the covariate as well as the Hessian matrix, estimated in E‐Surge, was used to simulate 50,000 new values of apparent survival (*ϕ*) for each value of the covariate. The mean and bootstrapped 95% CI were derived from all simulated values of *ϕ* in each scenario and future time period. The future time periods were *Near future* (2016–2040) and *Distant future* (2060–2085). *Near future* was selected to cover the immediate time period, where the influence of an improved status of cod would have larger impact on sprat, relative to later (Niiranen et al., 2013). The *Distant future* projections correspond to when climate change is projected to have a positive influence on sprat relative to cod and hence potentially represent a contrasting situation.

The *ϕ* values simulated under the currently targeted scenario (*Decrease* of nutrient input and *Precautionary* cod fishing pressure; hereafter targeted scenario) were each used in a matrix population model. Other parameters in the matrix, including reproductive success and immature survival, were selected within ranges reported in the literature and to match the estimate of current annual growth rate (see Appendix A). We used the R package *popbio* (Stubben & Milligan, 2007), to calculate the dominant eigenvalue of the matrix, which represents the asymptotic finite population growth rate *λ*. Mean and bootstrapped 95% CI were derived from these *λ* values to illustrate scenario impacts as well as uncertainty. See Data S1 for the R script developed for the analysis.

## 3 RESULTS

### 3.1 Survival models

The general model structure with most support modeled both survival and transitions as constant, but with differences between birds ringed the preceding season and those ringed in previous years, thereby accounting for transients (Tables 1, 2). There was some model uncertainty regarding structure for detection probabilities (Table 3). The highest ranked model did not include a difference between locations, but models with different survival at Kalken compared to *Other* had some support as well (Table 4).

**Table 1:** Result of model selection for survival of common murres at Kalken in the Stockholm archipelago, Baltic Sea 1995–2015

	np	Deviance	QAICc	ΔQAICc	Model
*ϕ*(*a*)	**43**	**4,894.21**	**3,350.89**	**0.00**	**1**
*ϕ*(*a* * Kalken + Other)	44	4,891.63	3,351.26	0.37	2
*ϕ*(site)	43	4,897.59	3,353.14	2.25	3
*ϕ*((*a* + *t*) * Kalken + Other)	63	4,861.10	3,371.22	20.33	4
*ϕ*(*t*)	61	4,874.68	3,375.99	25.10	5
*ϕ*(*a* * Kalken + *t* * Other)	60	4,882.86	3,379.31	28.42	6
*ϕ*((*a* + *t*) * Kalken + *t* * Other)	79	4,853.39	3,400.69	49.80	7
*ϕ*(*a* * *t*)	80	4,850.70	3,401.08	50.19	8
*ϕ*(*a* * *t* * Kalken + Other)	81	4,847.88	3,401.39	50.50	9
*ϕ*(*a* * *t* * Kalken + *t* * Other)	97	4,840.26	3,431.62	80.73	10

Note

Selection was based on QAICc (Akaike's information criterion corrected for lack of fit and sample size) keeping the same structure for transition, *ψ*(*a*), and detection probabilities, *p*(site * (*t*, period 2) + recov(.)). Site refers to breeding site and means that the two areas considered here: *Kalken* or *Other* (other locations within the Baltic Sea) is modeled independently. *a* indicates a transience model (Pradel, Hines, Lebreton, & Nichols, 1997), that is, an effect of “ringing age”—time since ringing as all birds in the study were ringed as full‐grown (in their second year or later); *t* time‐dependence and Other refers to birds recaptured at other locations in the Baltic Sea region. * refers to multiplicative effects, + to additive effects, and . to constant. np—number of parameters. The most supported model is indicated in bold.

**Table 2:** Result of model selection for transition probabilities, using the four most supported model structures for survival (see Table 1) and keeping the same model structure for detection probabilities, *p*(site * (*t*, period 2) + recov(.))

Survival	Transition	np	Deviance	QAICc	ΔQAICc	Model
*ϕ*(*a*)	***ψ*(*a*)**	**43**	**4,894.21**	**3,350.89**	**0.00**	**1**
*ϕ*(a * Kalken + Other)	*ψ*(.)	43	4,916.55	3,365.78	14.89	11
*ϕ*(*a*)	*ψ*(.)	42	4,925.70	3,369.78	18.89	12
*ϕ*((a + t) * Kalken + Other)	*ψ*(.)	62	4,886.74	3,386.17	35.28	13
*ϕ*(site)	*ψ*(.)	42	4,964.17	3,395.43	44.54	14
*ϕ*(*a*)	*ψ*(*a* * *t*)	80	4,847.93	3,399.24	48.35	15
*ϕ*(*a* * Kalken + Other)	*ψ*(*a* * *t*)	81	4,846.69	3,400.60	49.71	16
*ϕ*(site)	*ψ*(*a* * *t*)	80	4,854.01	3,403.28	52.39	17
*ϕ*((*a* + t) * Kalken + Other)	*ψ*(*a* * *t*)	99	4,814.86	3,419.15	68.26	18

Note

The model with most support from the previous selection stage (Model 1) is included for comparison. The most supported model is indicated in bold. For abbreviations, see Table 1.

**Table 3:** Result of model selection for detection probabilities, showing the results using the model structure with most support, for survival (*ϕ*(*a*), see Table 1) as well as transition probabilities (*ψ*(*a*), Table 2)

	np	Deviance	QAICc	ΔQAICc	Model
Time effects in recapture probabilities					
*p*(site * (*t*, period 2) + recov(.))	43	4,894.21	3,350.89	0	1
*p*(Kalken * (*t*, period 2) + Other * (period 1, period 2) + recov(.))	**26**	**4,915.93**	**3,330.05**	**−20.84**	**19**
*p*(recap(*t*, period 2) + recov(.))	24	4,932.56	3,337.03	−13.86	20
*p*(site * *t* + recov(.))	45	4,890.16	3,352.38	1.49	21
*p*(site * (period 1, period 2) + recov(.))	9	5,107.37	3,423.01	72.12	22
*p*(recap(period 1, period 2) + recov(.))	7	5,125.24	3,430.89	80.00	23
*p*(Kalken * (period 1, period 2) + Other * (*t*, period 2) + recov(.))	26	5,085.05	3,442.80	91.91	24
“Ringing age” effects in recapture at Kalken					
*p*(Kalken * (*t*, period 2) + Other * (period 1, period 2) + recov(.))	**26**	**4,915.93**	**3,330.05**	**0.00**	**19**
*p*(Kalken * (*a* * *t*, *a*. period 2) + Other * (period 1, period 2) + recov(.))	44	4,878.63	3,342.60	12.55	25
*p*(Kalken * ((*t*, period 2) + *a*) + Other * (period 1, period 2) + recov(.))	**27**	**4,911.96**	**3,329.46**	**−0.59**	**26**

Note

The model with most support in the previous modeling stage is included for comparison. The most supported model(s) in each stage is indicated in bold. For abbreviations, see Table 1.

**Table 4:** Summary of model selection for common guillemots at Kalken in the Stockholm archipelago, Baltic Sea

*Φ*	*Ψ*	*p*	np	Deviance	ΔQAICc
*a*	*a*	Kalken * ((*t*, period 2) + *a*) + Other * (period 1, period 2) + recov(.)	27	4,911.96	0.00
*a*	*a*	Kalken * (*t*, period 2) + Other * (period 1, period 2) + recov(.)	26	4,915.93	0.59
*a* * Kalken + Other	*a*	Kalken * ((*t*, period 2) + *a*) + Other * (period 1, period 2) + recov(.)	28	4,911.05	1.46
*a* * Kalken + Other	*a*	Kalken * (*t*, period 2) + Other * (period 1, period 2) + recov(.)	27	4,914.79	1.89
Site	*a*	Kalken * ((*t*, period 2) + *a*) + Other * (period 1, period 2) + recov(.)	27	4,917.89	3.96
Site	*a*	Kalken * (*t*, period 2) + Other * (period 1, period 2) + recov(.)	26	4,921.97	4.62

Note

Model structures within 7 QAICc units from the model with most support are listed. An *a* indicates a transience model (Pradel et al., 1997), with two “ringing age”‐classes, that is, an effect of time since ringing, separating birds ringed the preceding season and those ringed earlier; *t* indicates time‐dependence; and Site refers to breeding site and means that the two areas considered here: *Kalken* or *Other* (other locations in the Baltic Sea region) is modeled independently. Interactions are indicated by *, additive effects by +, while . indicates constant parameters.

### 3.2 Influence of prey and climate

**Figure 3:**
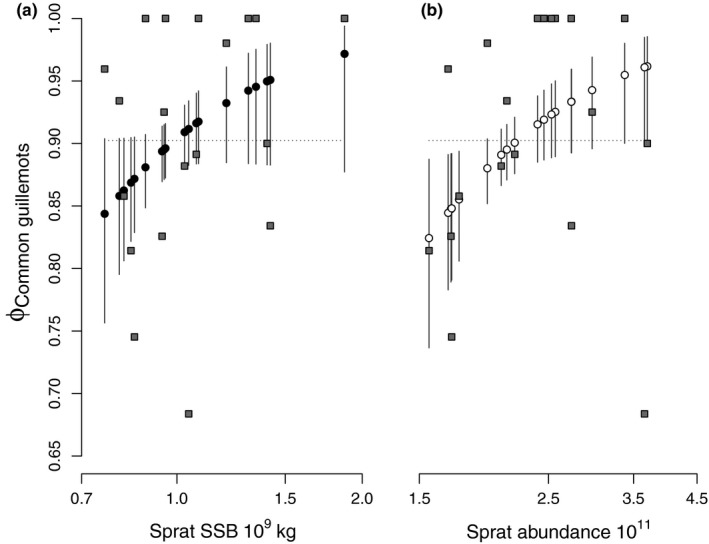
Survival of common guillemots *Uria aalge* at Kalken, Svenska högarna, Baltic Sea 1996–2015, estimated as a function of spawning stock biomass, SSB (a), and abundance (b) of their prey, sprat (*Sprattus sprattus*). The relationships are illustrated with circles, and solid lines show 95% CI. Gray squares indicate estimates from a model with time‐dependent survival but not containing any of the covariates. The dotted line illustrates the constant survival estimated by the model with most support among those that did not include any covariate

Prey quantity had significant impacts on survival rates of previously ringed guillemots (Table 5, Figure 3). The log‐transformed mean sprat weight at 4 years of age (a measure of prey quality) was strongly and negatively related to survival. We posit that this relationship was a consequence of the negative relationship between sprat quantity and quality rather than a reflection of a causal relationship (Casini et al., 2011; Österblom, Casini, Olsson, & Bignert, 2006). Therefore, we did not analyze this covariate further. Our models did not reveal any influence of climate when regional or local covariates were used (Table 5). Sprat abundance and SSB had a positive relationship with guillemot survival, but abundance explained more variation in survival, 34% of the total variation, than SSB did (Table 5). Nonlinear relationships (log‐transformed prey quantities) received more support than untransformed covariates did (Table 5).

**Table 5:** Analysis of deviance test results, including covariate tested, covariate model deviance, test results, *p*‐value, and R^2^, equivalent to a squared correlation coefficient, calculated based on differences in deviance between survival models with, and without, time‐dependence and with the covariate

Covariate	Deviance	ANODEV test	*p*	*R* ^2^ (%)
Sprat SSB	4,906.7	*F* = 5.2, *df*._cov_ = 1	0.036	23
log(Sprat SSB)	4,906.4	*F* = 5.6, *df*._cov_ = 1	0.030	25
Sprat abund	4,905.2	*F* = 7.4, *df*._cov_ = 1	0.015	30
log(Sprat abund)	4,904.4	*F* = 8.7, *df*._cov_ = 1	0.009	34
wNAO_0_	4,910.7	*F* = 1.0, *df*._cov_ = 1	0.34	–
wNAO_1_	4,911.7	*F* = 0.17, *df*._cov_ = 1	0.68	–
SST_0_	4,909.5	*F* = 2.5, *df*._cov_ = 1	0.16	–
SST_1_	4,911.5	*F* = 0.41, *df*._cov_ = 1	0.58	–
Ice cover_0_	4,910.1	*F* = 1.9, *df*._cov_ = 1	0.23	–
Ice cover_1_	4,910.7	*F* = 1.2, *df*._cov_ = 1	0.33	–

Note

Results indicate that survival of common guillemots at Kalken, Baltic Sea, was related to prey quantity but associated with climate.

Subscripted numbers indicate if climate variables were modeled with a time‐lag of one year (_1_) or without (_0_).

Abbreviations: abund, abundance in no. of individuals; SSB, Spawning stock biomass; SST, sea surface temperature January–March in the Baltic Sea; wNAO, North Atlantic Oscillation during winter (December–March).

### 3.3 Scenario analysis

**Figure 4:**
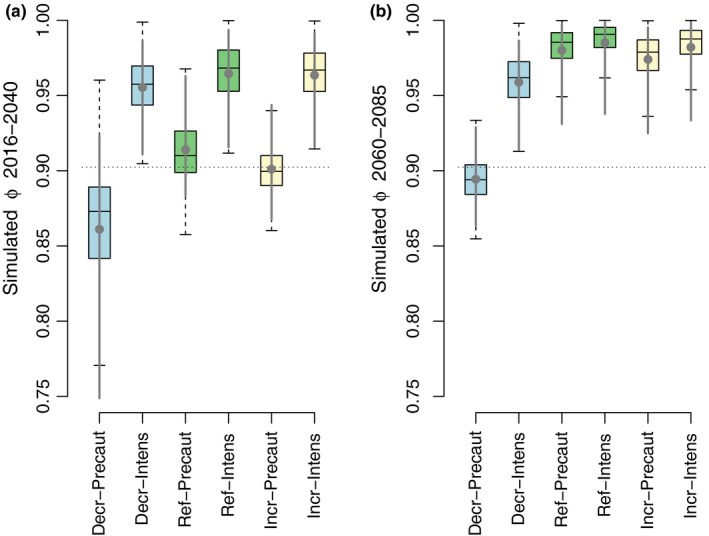
Survival rates of common guillemots *Uria aalge* at Kalken, Svenska Högarna, Baltic Sea under management scenarios for (a) 2016–2040 and (b) 2060–2085. The scenarios concerned the main regional drivers: eutrophication and cod fisheries, while incorporating climate change. *Increase*, *Decrease*, and *Reference* levels of nutrient inputs, as well as *Precautionary* versus *Intensive* cod fisheries, had been simulated in a food‐web model (Niiranen et al., 2013), from which estimates of sprat were used to project guillemot survival. Boxplots illustrate the medians and 50% of the projected values, and whiskers show approximate 95% CI for the medians. Gray dots denote the mean, and solid gray lines bootstrapped 95% CI. The dotted line illustrates the constant survival estimated by the model with most support among models without covariates

Using the relationship between survival rates of previously ringed guillemots and sprat SSB (sprat abundance could not be used in simulations because abundance projections were not available whereas SSB projections were), we projected survival under six scenarios. Simulated future survival of guillemots was higher or similar to current levels in all but one scenario: the targeted scenario (*Decr‐Precaut*; Figure 4). In the near future (2016–2040), mean survival was projected to increase in scenarios with *Intensive* cod fishing and remain similar to the current level under *Precautionary* cod fisheries combined with *Reference* levels or *Increase* of nutrient input (*Ref‐Precaut* and *Incr‐Precaut*; Figure 4a). The targeted scenario (i.e., *Precautionary* cod fishing and *Decreased* nutrient input) reduced mean guillemot survival to 0.86 (CI: 0.75–0.92, Figure 4a). Sprat projections and simulated guillemot survival were higher than current levels in the distant future (2060–2085) in all scenarios except the targeted scenario, where a minor decrease in survival (mean 0.894, CI: 0.86–0.93) was projected (Figure 4b).

**Figure 5:**
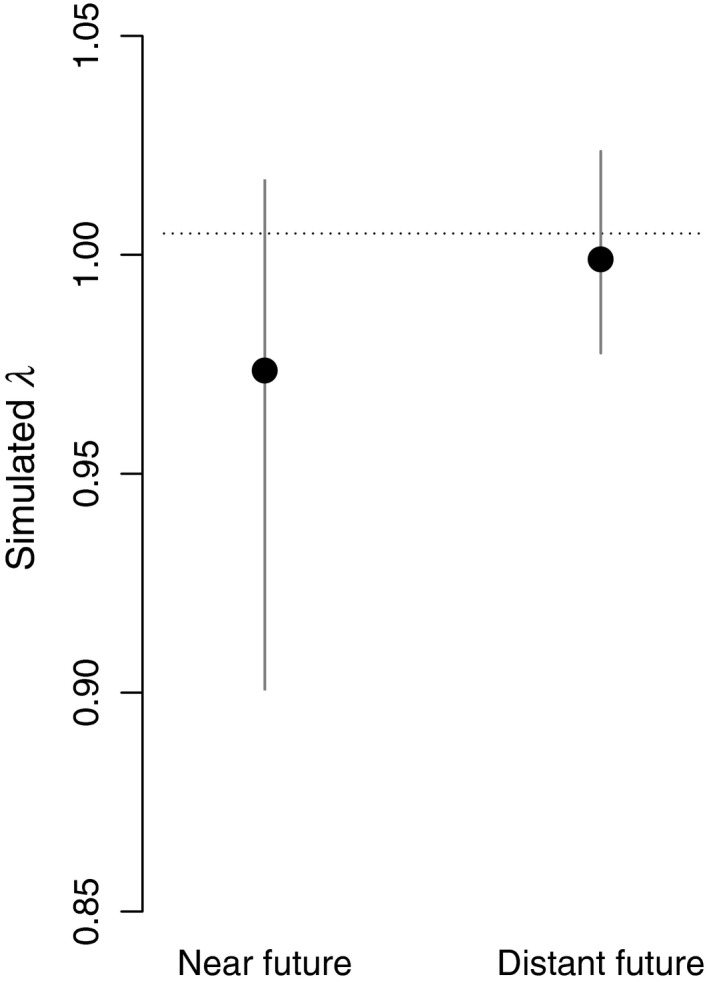
Simulated population growth rates, *λ*, of common guillemots at Kalken, Svenska Högarna, Baltic Sea. Matrix population models, with adult survival simulated under the *Precautionary* cod fishing and *Decreased* nutrient input scenario for the Baltic Sea, indicated a population growth rate substantially below 1 in the near future, 2016–2040, and a slightly negative growth rate in the distant future, 2060–2085. Points illustrate the mean and solid lines the 95% CIs. The dotted line shows the current *λ*, calculated from total population counts for Svenska Högarna (see Appendix A)

Negative population growth rates were projected when using simulated adult survival values from the targeted scenario (Figure 5). Matrix model projections suggested a substantial population decline during the near future: 24% over 10 (average) years, however a smaller reduction, 1.1%, over 10 years in the distant future. These numbers can be compared with counts from 1995–2015, which produced an annual growth rate estimate *λ* = 1.0049. This corresponds to a 5.0% increase over 10 years (using the more optimistic of available count data, see Appendix A).

## 4 DISCUSSION

Quantitative assessments of management alternatives are increasingly the standard of ecosystem‐based management for the oceans (Levin et al., 2009), but methods that can handle multiple management objectives are still rarely applied. Our study is one of the first to link demographic models with food‐web models (Figure 2) to understand specific impacts of management actions targeting broad‐scale challenges. Linking the modeling approaches can reveal synergies or, as in our case study, trade‐offs. We found that negative impacts on the survival and population growth rates of Baltic Sea guillemots are likely if the scenario mirroring current management initiatives, that is, *Precautionary* fishing to restore the cod stock and *Reduced* nutrient input to combat eutrophication, is successfully implemented, uncovering management objectives in conflict with each other.

### 4.1 Conservation of common guillemots in the Baltic Sea

The projected negative future trend is a contrast to the current favorable conservation status of the Baltic Sea guillemot population. Colonies have increased, and additional ones have become established during the last decades, which at least partly can be attributed to high prey abundance and lower bycatch rates following a ban of salmon drift nets (Olsson & Hentati‐Sundberg, 2017; Staav, 2009). A small decrease in one demographic rate may thereby not lead to a population decline, but the projected decrease in survival is substantial under the *Precautionary* cod fishing–*Reduced* nutrients scenario.

The *Precautionary* cod fishing–*Reduced* nutrients scenario can be regarded as an attempt to maximize cod, as the current level of eutrophication is considered harmful to the cod stock due to increased hypoxia (Casini et al., 2016; Hinrichsen et al., 2011). Considering the projected negative impacts on guillemots (Figure 5), it demonstrates a clear trade‐off between objectives to restore cod and reduce eutrophication, and the conservation of guillemots in this system.

The actual adverse impacts on the guillemot population may be smaller, however, despite current efforts to make the *Precautionary*–*Reduced* scenario a reality. The Baltic sprat stock is likely to increase under projected climate change (MacKenzie, Gislason, Möllmann, & Köster, 2007), and while climate change was incorporated in our scenarios, current CO_2_ emissions have followed the highest of the emission scenarios (Boden, Marland, & Andres, 2017; Manning et al., 2010). More substantial changes may give a relative advantage to sprat, and an increase in sprat may in turn benefit guillemots. Worth noting is that reduced fishing pressure on cod has so far not led to any detectable recovery of the cod stock (ICES, 2016), suggesting that lower predation pressure on sprat from a suppressed cod stock may continue into the future. Cod productivity appears to have been reduced in recent years, and in case, this is caused by a mechanism not accounted for in the food‐web model (e.g., a disease, change in behavior of predators or their prey); the cod recovery modeled under the *Precautionary*–*Reduced* scenario may be too optimistic. However, if lower cod productivity is related to environmental factors included in the food‐web model (e.g., hypoxia, as suggested by Casini et al. (2016)), or to changes in the availability of food resources on the Central Baltic Sea scale, the food‐web model should be able to account for this. In addition, our population model for guillemots assumes no changes in fecundity or prebreeding survival, or density‐dependent effects. Changes in breeding success related to quality of sprat (Kadin et al., 2012) and density dependence may dampen population impacts. For example, a smaller guillemot population may not be constrained by food limitation, resulting in relatively higher juvenile survival. However, the covariate that explained more variation in guillemot survival than any other we examined was sprat abundance, but we could not use abundance in simulations because abundance projections were not available whereas SSB projections were. Particularly if the future changes in abundance are more pronounced than changes in SSB, this would lead to impacts on guillemots that are potentially larger than projected.

Reliability of the future projections is also related to the time scales involved. The distant future projections (2060–2085) go substantially further into the future than the length of the data time series used to derive relationships. This implies that there is substantial uncertainty regarding specific outcomes. However, the potential for negative impacts on the guillemot population (Figure 5), even when climate change is projected to favor sprat, is essential to keep in mind when making decisions about management and monitoring.

If negative impacts on guillemots were detected, there would be several strategies with potential to mitigate effects without compromising the objectives of cod recovery and reduced nutrient input. Minimizing local competition with fisheries and continued efforts to remove the nest predator American mink *Neovison vison* would help ensure successful reproduction. Other sources of mortality can be reduced by, for example, additional bycatch mitigation efforts. Direct and indirect effects of white‐tailed eagles *Haliaeetus albicilla*, via disturbance and predation, may be monitored and can perhaps be alleviated.

### 4.2 Integration of apex predator conservation and ecosystem‐based management

Uncovering conflicting objectives is an essential but challenging aspect of evaluating ecosystem management alternatives. Eutrophication with associated hypoxia and algal blooms, high exploitation rates, and suppressed populations of apex predators are issues far from unique to the Baltic Sea, but central to managers worldwide (Lotze et al., 2006). Predicting the net outcomes of management interventions targeting these issues is not straightforward, and most studies focus on the stocks that are directly impacted, often commercially harvested fish (Fu et al., 2018). While food‐web models can have high taxonomic resolution also for indirectly affected predators (Koehn et al., 2017), this is rarely implemented. We have demonstrated that approaches linking existing food‐web models with demographic models have the potential to reveal net effects on different species of interest. Relevant monitoring data are available in many cases that, when analyzed with demographic models, would enable population‐specific responses to be quantified, thus illustrating effects of management alternatives at the same resolution for apex predators as for fish.

Specific and quantified impacts on indirectly impacted populations can be as important as information about direct effects when selecting large‐scale management measures. Quantification makes comparisons straightforward and can include illustrations of uncertainty regarding outcomes. This knowledge is fundamental to explicit discussions about trade‐offs, which is a central component of transparent and deliberative decision‐making (Gregory et al., 2012). Predictions of indirect effects on species such as guillemots will rarely be obtainable from food‐web models or demographic models in isolation.

While conservation objectives may be the most obvious reason for linking demographic and food‐web models, concerns over potential pests or invasive species could be other reasons to use the approach. Potential population trends can be explored to provide insights on future risk and the need to take further action. Additionally, linked approaches can include top‐down effects, such as predation or trophic cascades, as well as bottom‐up effects, whereby different management measures with similar impacts, qualitatively or quantitatively, can be detected. Such results assist in finding cost‐effective measures, irrespective of whether the concern is a population increase or decline.

### 4.3 Tailoring approaches linking demographic and food‐web models

Our work demonstrates a likely trade‐off for ecosystem management in the Baltic Sea, between high‐trophic level fish, reduced eutrophication, and conservation of seabirds. While the approach can be transferred to other ecosystems in its current format, additional refinement would increase its relevance. Increasing model complexity when data are available could improve predictive power. The matrix population model we used does not account for potential density dependence. Integrated population models, which jointly model different streams of demographic data, would allow for simultaneous modeling of survival, reproduction, and transitions as a function of environmental or other covariates (Abadi, Gimenez, Arlettaz, & Schaub, 2010) as well as density dependence (Schaub, Jakober, & Stauber, 2013). Such improvements would allow for more realistic relationships with drivers to be modeled. Another expansion would involve integration of an age‐ or stage‐structured fish stock model, which could simulate proxies of prey quantity and quality based on climate projections and food‐web model outputs (see Bartolino et al., 2014 for an example). The outputs from such a model would allow prey quality to be represented. This could be especially relevant for making projections for our study species and other apex predators dependent on quality in addition to quantity (Österblom, Olsson, Blenckner, & Furness, 2008).

Direct coupling of the demographic and the food‐web models would be an advantage when expecting top‐down effects of the species of concern, for example, a pest species. Our models were linked to incorporate bottom‐up effects on guillemots, but do not include a top‐down effect on sprat in turn. The abundance of many seabirds feeding on schooling pelagic fish (such as sprat) is generally thought to be bottom‐up controlled by prey availability, and they often require a much larger prey base than their actual energy needs (Cury et al., 2011). As a consequence, their consumption of, for example, sprat is much smaller than that of fish predators and fisheries (Engelhard et al., 2013; Hansson et al., 2017), and any impact of guillemots on sprat abundance is likely to be small. However, for other species or ecosystems, such as coastal systems, impacts may be larger (Hansson et al., 2017) and require direct coupling to accurately capture dynamics.

### 4.4 Policy implications

Policy frameworks that seek to balance diverse interests, such as ecosystem‐based management, could better serve those aims by explicitly using integrated analysis approaches when possible. Iterative evaluations of management alternatives and a focus on the short term may allow ecological forecasts (Dietze et al., 2018), in addition to scenario analysis, to inform decisions.

Assessments of current status and management alternatives are typically based on ecological indicators, directly measured or derived from models (Levin et al., 2009). Conflicting objectives may, when not accounted for, complicate the use and interpretation of indicators. As follows from our case study, a decline of forage fish consumers such as seabirds is not necessarily a sign of an ecosystem in poor health, and it may signal development toward an oligotrophic ecosystem with abundant predatory fish. If maintaining seabird populations has been set as a standard for acceptable environmental status, along with, for example, an oligotrophic status and abundant predatory fish, an acceptable status of guillemots will be very challenging to fully achieve (cf. EU Directive 2008/56/EC; Reilly, Fraser, Fryer, Clarke, & Greenstreet, 2013; HELCOM, 2018). Management efforts will thus be perceived as only partially successful. Rather, the definitions of indicator target levels and decision thresholds that trigger management action (Martin, Runge, Nichols, Lubow, & Kendall, 2009) will be more realistic if they are set in recognition of trade‐offs between objectives. Decision thresholds can be viewed as functions of management objectives as well as of ecological thresholds, and a clear distinction between the subjective (the management objectives and their prioritization) and objective (ecosystem structure and state) components allows for structured decision‐making (Martin et al., 2009), reducing the risk of aiming for objectives that are not simultaneously achievable. Conflicting objectives are thus essential to consider not only when deciding on management actions, but also when designing the mechanisms to evaluate their success.

## 5 CONCLUSIONS

By linking a demographic and a food‐web model, we illustrate an approach for uncovering trade‐offs or synergies between management objectives. The case study incorporates common objectives in marine ecosystem‐based management: high‐trophic‐level fish of interest to commercial fisheries, minimized impacts of eutrophication, and conservation of fish‐dependent species. With the necessary data and underlying models readily available in many ecosystems, this approach enables inclusion of objectives that traditionally have received little attention in decision‐making processes. Linked approaches facilitate comparison and ranking of alternatives, which make priorities transparent. Conflicting objectives will be inherent in management of any ecosystem, but integration of modeling techniques allows for better‐informed decisions when aiming to balance diverse interests and drivers of change.

## CONFLICT OF INTEREST

None declared.

## AUTHOR CONTRIBUTIONS

MK, MF, SN, and SJC conceived the ideas and designed the methodology; MK and SJC analyzed the data; and MK led the writing of the manuscript. All authors contributed critically to the drafts and approved publication.

## Data Availability

Data are available from the Dryad Digital Repository https://doi.org/10.5061/dryad.b5n8220.

## APPENDIX A

### ADDITIONAL DETAIL ON MATERIAL AND METHODS

#### MULTISTATE MODEL STRUCTURE

The data were modeled in a multistate framework using E‐Surge 1.8.5 (Choquet, Rouan, et al., 2009). Birds ringed at Kalken were also recaptured or resighted elsewhere—mainly on other Stockholm archipelago islands, but also reported from other colonies in the Baltic Sea (*n*_Individuals_ = 38). Therefore, we used two “Alive” states to reduce the bias that emigration otherwise would have caused. We also included a “Newly dead” state to further reduce bias. However, finding dates for birds found dead and reported to the Swedish Bird Ringing Centre were often uncertain resulting in only 4 dead recoveries included in the analysis.

We thus used four states in the models: Alive at Kalken, Alive Other, Newly Dead and Dead. The following matrix patterns were used in the E‐Surge models:

We used four detection events:

0. Not encountered,
1. Captured or recaptured at Kalken
2. Recaptured or resighted at other colonies
3. Found dead.

#### MODEL SELECTION

Initial state probabilities were not explicitly modeled since all birds were first captured at Kalken and hence in state 1. We evaluated all relevant models for survival probabilities, but transition probabilities (contingent on survival) were restricted: The models allowed for transitions from Kalken to other places but not from Other back to Kalken. There was one individual that had an encounter history with one observation Other in between several recaptures at Kalken. Here, we opted for treating the single Other observation as “Not encountered” in the analysis since a full assessment of transitions between colonies was beyond the scope of the study.

Recapture and resighting probabilities were modeled as time‐dependent, constant 1996–2013 (period 1) or constant 2014–2015 (period 2) because one ringer was responsible for field work up to 2012 and a new ringer took over in 2013, from his second year (2014) substantially increasing effort and efficiency of ringing activities. Recovery probabilities were assumed constant. Survival, transition, and resighting probabilities at Kalken were also modeled with and without “ringing age”‐dependence (time since ringing, using two age classes) to account for transience effects. Birds in state Other were in the second “ringing age”‐class, so “ringing age” was not relevant. We assumed that dead birds had the same chances of being found and reported regardless of time since ringing.

We checked how rankings of the models changed when increasing $\hat{c}$ from 1.5. With *ĉ* = 1.65, the ranking remained the same for the top three models. At 2.0 and 2.5, the five highest ranked models remained the top five, but the individual order changed slightly, with the highest ranked model at $\hat{c}$ = 1.5 being ranked second.

#### POPULATION SIMULATIONS

Common murres have been counted annually at Kalken, as well as within the small group of islands, Svenska Högarna, that Kalken is part of. We choose to use the Svenska Högarna counts 1995 (*n* = 490) and 2015 (*n* = 540) to get a rough estimate of the population trend, as the overall Svenska Högarna trend was slightly positive and would thereby give us an somewhat optimistic baseline. In contrast, Kalken alone had a negative trend over the study period and using this estimate would increase the chance of finding negative impacts of future scenarios on population growth rates. Count data were provided by the Archipelago Foundation (The Archipelago Foundation, 2016).

A female‐based population matrix *A* with five age classes, of which the adult age class is reproducing

was parameterized to match the Svenska Högarna population trend of *λ* = 1.0049, using *ϕ*_Kalk_ from the selected survival model and immature survival *ϕ*_1–4_ as well as breeding success within ranges reported in the literature (Crespin, Harris, Lebreton, Frederiksen, & Wanless, 2006; Harris, Frederiksen, & Wanless, 2007; Votier et al., 2008; Wanless, Harris, Redman, & Speakman, 2005). Because of the postbreeding census, we modeled *F* = *ϕ*_Kalk_ ***** *b*. The parameter values subsequently used in simulations were as follows: *ϕ*_1_ = 0.62, *ϕ*_2_ = 0.70, *ϕ*_3_ = 0.80, *ϕ*_4_ = 0.87, and *b* = 0.385 (corresponding to a breeding success of 0.77 offspring/pair and a 1:1 sex ratio). These values were assumed constant throughout the simulations.
