# Supplementary material for: Linking demographic and food‐web models to understand management trade‐offs
Source: Ecol Evol. 2019 Jul 17;9(15):8587–600. doi: 10.1002/ece3.5385 (PMC6686646; doi:10.1002/ece3.5385)
Supplement: Supplementary file 1 [file ECE3-9-8587-s001.docx]

#####################################################################

## Scenario simulations

## * Survival at Kalken under six future scenarios

## * Population growth rate under most desired scenario

## Authors: Martina Kadin and Sarah J Converse

#####################################################################

# ~~~~~~~~~~~~~~~~~~~~~~~~~~~~~~~~~~~~~~~~~~~~~~~~~~~~~~~~~~~~~~~~~~~~~

# ~~~~~~~~~~~~~~~~~~~~~~~~~~~~~~~~~~~~~~~~~~~~~~~~~~~~~~~~~~~~~~~~~~~~~

#Input data

#################

library(gdata) #needed to load xls files

#################

#Datasets with survival estimates from E-Surge

#################

# spr.ssb.real <- read.xls("/Users/User/Documents/Kalken_Survival_E-

# SurgeResults/phi(a+a2.SprSSB-ln)psi(a)p(Kalk.((t,per2)+a)+Else.(per1,per2)+recov).xls",

# sheet="Reduced Set of Parameters")

# constant <- read.xls("/Users/User/Documents/Kalken_Survival_E-

# SurgeResults/phi(a)psi(a)p(Kalk.((t,per2)+a)+Else.(per1,per2)+recov).xls",

# sheet="Reduced Set of Parameters")

#################

#Beta estimates from E-Surge

#################

# spr.ssb.beta <- read.xls("/Users/User/Documents/Kalken_Survival_E-

# SurgeResults/phi(a+a2.SprSSB-ln)psi(a)p(Kalk.((t,per2)+a)+Else.(per1,per2)+recov).xls",

# sheet="Beta")

#################

#Hessian estimates from E-Surge

#################

# spr.ssb.hess <- read.xls("/Users/User/Documents/Kalken_Survival_E-

# SurgeResults/phi(a+a2.SprSSB-ln)psi(a)p(Kalk.((t,per2)+a)+Else.(per1,per2)+recov).xls",

# sheet="Hessian", header = FALSE)

spr.ssb.hess.f <- spr.ssb.hess[1:3,1:3] #Selecting the relevant section of the hessian

spr.ssb.hess <- spr.ssb.hess.f[c(2,3),c(2,3)] #Selecting the relevant values

#################

#Datasets with scenario sprat data from BaltProWeb

#################

# scen.org <- read.xls("/Users/User/Documents/CovariateData/SpratData_EwE_170816.xlsx")

# ~~~~~~~~~~~~~~~~~~~~~~~~~~~~~~~~~~~~~~~~~~~~~~~~~~~~~~~~~~~~~~~~~~~~~

# ~~~~~~~~~~~~~~~~~~~~~~~~~~~~~~~~~~~~~~~~~~~~~~~~~~~~~~~~~~~~~~~~~~~~~

# ~~~~~~~~~~~~~~~~~~~~~~~~~~~~~~~~~~~~~~~~~~~~~~~~~~~~~~~~~~~~~~~~~~~~~

#Simulation settings

start.near <- 2016 #Start year for near future scenario runs

end.near <- 2040 #End year for near future scenario runs

start.far <- 2060 #Start year for distant scenario runs

end.far <- 2085 #End year for distant scenario runs

# ~~~~~~~~~~~~~~~~~~~~~~~~~~~~~~~~~~~~~~~~~~~~~~~~~~~~~~~~~~~~~~~~~~~~~

# ~~~~~~~~~~~~~~~~~~~~~~~~~~~~~~~~~~~~~~~~~~~~~~~~~~~~~~~~~~~~~~~~~~~~~

# ~~~~~~~~~~~~~~~~~~~~~~~~~~~~~~~~~~~~~~~~~~~~~~~~~~~~~~~~~~~~~~~~~~~~~

#Simulations

# ~~~~~~~~~~~~~~~~~~~~~~~~~~~~~~~~~~~~~~~~~~~~~~~~~~~~~~~~~~~~~~~~~~~~~

# ~~ Predictions with noise (resampling from Hessian), near future ~~~

# ~~~~~~~~~~~~~~~~~~~~~~~~~~~~~~~~~~~~~~~~~~~~~~~~~~~~~~~~~~~~~~~~~~~~~

#Data frame to fill with values

dd.scen <- scen[which(scen$year>=start.near & scen$year<=end.near),]

colnames(dd.scen)[2:7] <- c("Ref-F1.1", "Inc-F1.1", "Dec-F1.1", "Ref-F0.3", "Inc-F0.3", "Dec-F0.3")

#Intercept from E-Surge model

alfa <- spr.ssb.beta[2, "Value"]

#Slope from E-Surge model

beta <- spr.ssb.beta[3, "Value"]

estimate <- as.vector(c(alfa,beta))

hessian <- as.matrix(spr.ssb.hess)

hold <- estimate + t(chol(solve(hessian)))%*%matrix(rnorm(100000),ncol=50000)

result <- data.frame(Scen=rep(c("Ref-F1.1", "Inc-F1.1", "Dec-F1.1", "Ref-F0.3", "Inc-F0.3", "Dec-F0.3"), each=50000*nobs(dd.scen)))

result$SimSprat <- NA

for (scena in 2:7){

sprat <- dd.scen[,scena]

a2.survival <- matrix(NA,nrow=50000,ncol=nobs(dd.scen)[1])

res.temp <- rep(NA,times=50000*nobs(dd.scen)[1])

for(i in 1:50000){

for(s in 1:nobs(dd.scen)[1]){

a2.survival[i,s] <- 1/(1+exp(-(hold[1,i] + hold[2,i]*log(sprat[s]))))

}

}

for(s in 1:nobs(dd.scen)[1]){

res.temp[(50000*(s-1)+1):(50000*s)] <- a2.survival[,s]

}

result[(1250000*(scena-2)+1):(1250000*(scena-1)), "SimSprat"] <- res.temp

}

################

# ~~~~~~~~~~~~~~~~~~~~~~~~~~~~~~~~~~~~~~~~~~~~~~~~~~~~~~~~~~~~~~~~~~~~~

# ~~ Predictions with noise (resampling from Hessian), far future ~~~~

# ~~~~~~~~~~~~~~~~~~~~~~~~~~~~~~~~~~~~~~~~~~~~~~~~~~~~~~~~~~~~~~~~~~~~~

#Data frame to fill with values

dd.scen <- scen[which(scen$year>=start.far & scen$year<=end.far),]

colnames(dd.scen)[2:7] <- c("Ref-F1.1", "Inc-F1.1", "Dec-F1.1", "Ref-F0.3", "Inc-F0.3", "Dec-F0.3")

#Intercept from E-Surge model

alfa <- spr.ssb.beta[2, "Value"]

#Slope from E-Surge model

beta <- spr.ssb.beta[3, "Value"]

estimate <- as.vector(c(alfa,beta))

hessian <- as.matrix(spr.ssb.hess)

hold <- estimate + t(chol(solve(hessian)))%*%matrix(rnorm(100000),ncol=50000)

result.far <- data.frame(Scen=rep(c("Ref-F1.1", "Inc-F1.1", "Dec-F1.1", "Ref-F0.3", "Inc-F0.3", "Dec-F0.3"), each=50000*nobs(dd.scen)))

result.far$SimSprat <- NA

for (scena in 2:7){

sprat <- dd.scen[,scena]

a2.survival <- matrix(NA,nrow=50000,ncol=nobs(dd.scen)[1])

res.temp <- rep(NA,times=50000*nobs(dd.scen)[1])

for(i in 1:50000){

for(s in 1:nobs(dd.scen)[1]){

a2.survival[i,s] <- 1/(1+exp(-(hold[1,i] + hold[2,i]*log(sprat[s]))))

}

}

for(s in 1:nobs(dd.scen)[1]){

res.temp[(50000*(s-1)+1):(50000*s)] <- a2.survival[,s]

}

result.far[((1300000*(scena-2)+1):(1300000*(scena-1))), "SimSprat"] <- res.temp

}

################

############

#Boxplots of near and far scenarios

###########

plotd.near <- result

plotd.near$Period <- "Near future"

plotd.far <- result.far

plotd.far$Period <- "Far future"

#Code to calculate and plot mean and CIs

quant25 <- function(x) quantile(x, probs=0.025)

quant975 <- function(x) quantile(x, probs=0.975)

tes <- as.vector(tapply(plotd.near$SimSprat, list(plotd.near$Scen), FUN=quant25))

cis <- data.frame(ci.l=tes)

tes <- as.vector(tapply(plotd.near$SimSprat, list(plotd.near$Scen), FUN=quant975))

cis$ci.u <- tes

tes <- as.vector(tapply(plotd.near$SimSprat, list(plotd.near$Scen), FUN=mean))

cis$mean <- tes

cis$Scen <- levels(plotd.near$Scen)

fars <- as.vector(tapply(plotd.far$SimSprat, list(plotd.far$Scen), FUN=quant25))

cis.f <- data.frame(ci.l=fars )

fars <- as.vector(tapply(plotd.far$SimSprat, list(plotd.far$Scen), FUN=quant975))

cis.f$ci.u <- fars

fars <- as.vector(tapply(plotd.far$SimSprat, list(plotd.far$Scen), FUN=mean))

cis.f$mean <- fars

cis.f$Scen <- levels(plotd.far$Scen)

quartz()

par(mfrow=c(1,2))

boxplot(plotd.near$SimSprat ~ plotd.near$Scen, col=c("lightblue","lightblue","palegreen3", "palegreen3","lemonchiffon","lemonchiffon"),

frame=F, cex.axis=0.8, boxwex=0.5, medlwd=0.9, outline=F, ylim=c(0.75,1), axes=F,

ylab=expression(paste("Simulated ", phi, " 2016-2040")))

axis(2, yaxp=c(0.75,1,5))

axis(1, at=c(1:6), labels=c("Decr-Precaut", "Decr-Intens", "Ref-Precaut","Ref-Intens", "Incr-Precaut", "Incr-Intens"), cex.axis=0.8, las=2)

segments(x0=0.6, x1=6.4, y0=constant[which(constant$Age==2 & constant$Parameters==" Surv"), "Estimates"], col="grey30", lwd=2)

for (u in 1:6) {

segments(x0=u, x1=u, y0=cis$ci.l[u], y1=cis$ci.u[u], lwd=2, col="grey50")

points(x=u, y=cis$mean[u], pch=19, col="grey50")}

boxplot(plotd.far$SimSprat ~ plotd.far$Scen, col=c("lightblue","lightblue","palegreen3", "palegreen3","lemonchiffon","lemonchiffon"),

frame=F, cex.axis=0.8, boxwex=0.5, medlwd=0.9, outline=F, ylim=c(0.75,1), axes=F,

ylab=expression(paste("Simulated ", phi, " 2060-2085")))

axis(2, yaxp=c(0.75,1,5))

axis(1, at=c(1:6), labels=c("Decr-Precaut", "Decr-Intens", "Ref-Precaut","Ref-Intens", "Incr-Precaut", "Incr-Intens"), cex.axis=0.8, las=2)

segments(x0=0.6, x1=6.4, y0=constant[which(constant$Age==2 & constant$Parameters==" Surv"), "Estimates"], col="grey30", lwd=2)

for (u in 1:6) {

segments(x0=u, x1=u, y0=cis.f$ci.l[u], y1=cis.f$ci.u[u], lwd=2, col="grey50")

points(x=u, y=cis.f$mean[u], pch=19, col="grey50")}

# ~~~~~~~~~~~~~~~~~~~~~~~~~~~~~~~~~~~~~~~~~~~~~~~~~~~~~~~~~~~~~~~~~~~~~

# ~~~~~~~~~~~~~~~~~~~~~~~~~~~~~~~~~~~~~~~~~~~~~~~~~~~~~~~~~~~~~~~~~~~~~

# ~~~~~~~~~~~~~~~~~~~~~~~~~~~~~~~~~~~~~~~~~~~~~~~~~~~~~~~~~~~~~~~~~~~~~

#Life history parameters and matrix models, in scenario Decr-Precaut

library(popbio)

#Mean values in near and distant future periods, in scenario Decr-Precaut

mean.near <- mean(result[which(result$Scen=="Dec-F0.3"),"SimSprat"])

mean.far <- mean(result.far[which(result.far$Scen=="Dec-F0.3"),"SimSprat"])

#Baseline, based on best available current information

classes<-c("1yr", "2yr", "3yr", "4yr", "Adults")

lambda.curr.counts <- (540/490)^(1/20) #Development over 20 years, from 490 to 540 counted.

curr.10yrs <- lambda.curr.counts^10

#Current situation

curr.phi <- 0.902

curr <- matrix(c(

0, 0, 0, 0, 0.385*curr.phi, #Female-offspring only

0.62, 0, 0, 0, 0,

0, 0.7, 0, 0 ,0,

0, 0, 0.8, 0, 0,

0, 0, 0, 0.87, curr.phi), nrow=5, byrow=TRUE, dimnames=list(classes,classes))

eigen.analysis(curr, zero=TRUE)

lambda(curr)

#Comparison current lambda based on counts and matrix model

lambda.curr.counts - lambda(curr)

##############

##############

#Analysis using the all projected phi-values

#Near future

allnear <- result[which(result$Scen=="Dec-F0.3"),"SimSprat"]

near.lambdas <- rep(NA, times=length(allnear))

for (w in 1:length(allnear)){

nearfut <- matrix(c(

0, 0, 0, 0, 0.385*mean.near, #Female-offspring only

0.62, 0, 0, 0, 0,

0, 0.7, 0, 0 ,0,

0, 0, 0.8, 0, 0,

0, 0, 0, 0.87, allnear[w]), nrow=5, byrow=TRUE, dimnames=list(classes,classes))

near.lambdas[w] <- lambda(nearfut)

}

#Distant future

allfar <- result.far[which(result.far$Scen=="Dec-F0.3"),"SimSprat"]

far.lambdas <- rep(NA, times=length(allfar))

for (w in 1:length(allfar)){

farfut <- matrix(c(

0, 0, 0, 0, 0.385*mean.far, #Female-offspring only

0.62, 0, 0, 0, 0,

0, 0.7, 0, 0 ,0,

0, 0, 0.8, 0, 0,

0, 0, 0, 0.87, allfar[w]), nrow=5, byrow=TRUE, dimnames=list(classes,classes))

far.lambdas[w] <- lambda(farfut)

}

#Population change over 10 years of average change in each simulation period

near.10yrs <- (mean(near.lambdas))^10

far.10yrs <- (mean(far.lambdas))^10

# ~~~~~~~~~~~~~~~~~~~~~~~~~~~~~~~~~~~~~~~~~~~~~~~~~~~~~~~~~~~~~~~~~~~~~
